# Comparative pangenomics of the *Corynebacterium kroppenstedtii* complex reveals species structure and accessory genome diversity

**DOI:** 10.3389/fmicb.2026.1947083

**Published:** 2026-09-07

**Authors:** Xihua Zhou, Yang Zhang, Yifan Qiu, Li Feng

**Affiliations:** Jiyang College, Zhejiang A&F University, Zhuji, Zhejiang, China

**Keywords:** antimicrobial resistance, *Corynebacterium kroppenstedtii* complex, mastitis, mobile genetic elements, pangenome, population genomics

## Abstract

**Background:**

The *Corynebacterium kroppenstedtii* complex (CKC) is increasingly recovered from mastitis, but its species boundaries and genomic diversity remain poorly resolved.

**Methods:**

Following quality screening, we analysed 56 publicly available genomes of *C. kroppenstedtii* (Ck), *C. parakroppenstedtii* (Cpak), and *C. pseudokroppenstedtii* (Cpse). Taxonomic assignments were supported by average nucleotide identity (ANI), digital DNA–DNA hybridization (dDDH), average amino acid identity (AAI)-derived protein similarity, and a core-genome-based phylogeny. We then examined pangenome structure, mobile elements, resistance genes, functional enrichment, and conserved gene neighbourhoods.

**Results:**

ANI and core-gene phylogeny resolved Ck, Cpak, and Cpse as three species. The 4,706-cluster pangenome was open (fitted exponent = 0.232) and comprised 1,751 core and 2,955 accessory clusters. Accessory-gene profiles further resolved three Cpak lineages, Cpak-P1 to Cpak-P3, concordant with the core-genome phylogeny. Insertion-sequence counts correlated with accessory cluster counts (Spearman’s *ρ* = 0.640, *p* = 1.12 × 10^−7^), and accessory clusters more frequently overlapped predicted mobile genetic elements (MGEs) than core clusters (402/2,955 versus 98/1,751; odds ratio = 2.66, *p* = 3.31 × 10^−19^), supporting an MGE–accessory association without implying causality. Seven ARG types were detected, most commonly *ermX*, *cmx*, *APH(6)-Id*, and *tet(W)*. AC410-like sequences occurred in 35/56 genomes and were strongly associated with antimicrobial-resistance gene (ARG) carriage (34/35 versus 4/21; odds ratio = 120.5, *p* = 9.93 × 10^−10^). In five of eight complete genomes, AC410-like sequences were located on the chromosome rather than on independent plasmid contigs. Functional enrichment and conserved gene neighbourhoods identified a candidate lipid–iron locus shared by Cpak-P1 and Cpak-P2.

**Conclusion:**

These findings provide a lineage-resolved genomic framework for the CKC, demonstrating an open pangenome with substantial species- and lineage-associated accessory diversity. The results further identify AC410-like resistance-associated genomic backgrounds and a candidate lipid–iron locus for future functional investigation.

## Introduction

*Corynebacterium kroppenstedtii* (Ck) is an infrequently reported, lipophilic corynebacterium that lacks mycolic acids and was first described in 1998 (Collins et al., 1998). Although the type strain originated from sputum, most reported infections have involved breast specimens from women with non-lactational mastitis, granulomatous inflammation, or recurrent abscesses (Paviour et al., 2002; Dobinson et al., 2015; Fernández-Natal et al., 2016). Patients may present with painful breast masses and undergo repeated drainage or surgery (Dobinson et al., 2015). In a retrospective cohort using the legacy Ck label, Ck-positive granulomatous mastitis was associated with more sinus formation and recurrence than culture-negative disease, although this finding does not establish CKC organisms as a universal cause of granulomatous mastitis (Li et al., 2023). Their recognition can nevertheless be difficult because lipophilic growth requirements and limited species resolution complicate recovery and identification in routine practice (Tauch et al., 2008; Luo et al., 2022).

The organisms historically reported as Ck are not genetically uniform. Genomic and phenotypic analyses of mastitis-associated isolates led to the description of *C. parakroppenstedtii* (Cpak) and *C. pseudokroppenstedtii* (Cpse), which together with Ck form the *C. kroppenstedtii* complex (CKC) (Luo et al., 2022). These species are difficult to distinguish using routine identification methods, raising uncertainty about which CKC member was represented in earlier clinical and genomic studies (Luo et al., 2022; Huang et al., 2024). A recent species-resolved series of severe non-lactational mastitis found that Cpak predominated among cultured CKC isolates, although the sample was too small to establish prevalence (Liang et al., 2024). Genomic studies have begun to explain the biology behind this clinical niche. Gene loss affecting mycolic-acid and fatty-acid biosynthesis, together with retention of beta-oxidation, provides a basis for the lipophilic lifestyle of Ck (Tauch et al., 2008). Comparisons of clinical genomes revealed substantial differences in acquired resistance determinants between isolates (Fernández-Natal et al., 2016). More recently, Cpak was shown to secrete an iron-chelating glycolipid that damaged mammary cells and promoted inflammatory responses in experimental systems (Liu et al., 2024). This mechanism is compelling but has been demonstrated for Cpak and cannot yet be generalized across the CKC.

These advances leave a broader question unresolved: how is functional diversity distributed across the CKC after its species boundaries are corrected? Much of the available evidence comes from individual isolates, small strain collections, or studies that used the legacy Ck label. The distribution of accessory genes involved in antimicrobial resistance, MGEs, lipid handling, iron acquisition, and host interaction therefore remains poorly resolved. A pangenome framework can distinguish functions conserved across the complex from those associated with particular species or within-species groups (Rouli et al., 2015; Li et al., 2025). In an open bacterial pangenome, continued sampling reveals additional gene families through the combined effects of population structure, ecological variation, gene gain and loss, and horizontal exchange (McInerney et al., 2017; Brockhurst et al., 2019). MGEs can contribute resistance and other accessory functions (Khedkar et al., 2022), but an association between MGE burden and gene content does not establish that they cause pangenome openness. Lineage composition, genome size, annotation yield, and assembly fragmentation can shape the same signals.

Against this background, we re-evaluated the taxonomy and genome content of 56 quality-screened CKC assemblies. Species boundaries were established using ANI and phylogenomics before pangenome inference, reducing dependence on historical database labels. We then examined accessory genome structure, within-Cpak genomic groups, MGE profiles, and ARG carriage. Two regions were examined in greater detail: an AC410-like resistance-associated region and a variably distributed locus annotated for lipid modification, export, and iron handling. By integrating species assignment, gene presence–absence patterns, functional annotation, and genomic context, we sought to define the major axes of CKC diversity. The analysis was designed to separate robust comparative-genomic patterns from hypotheses about mobility and biological function that still require experimental validation.

## Materials and methods

### Genome collection, quality control, and species assignment

An overview of the study workflow is provided in Supplementary Figure S1. We retrieved GenBank assemblies labelled as members of the CKC together with their assembly and BioSample metadata. Species assignments were evaluated against Ck DSM 44385 (NCBI accession: GCF_000023145.1), Cpak MC-26 (GCF_019754935.1), and Cpse P15_C1 (GCF_030486685.2). ANI was calculated with fastANI v1.34 (Jain et al., 2018), and assemblies below 95% ANI to every reference were excluded. Assembly quality was assessed with QUAST v5.3.0 and summarized with MultiQC v1.35 (Gurevich et al., 2013; Ewels et al., 2016). Assemblies combining less than 70% reference coverage with severe fragmentation were also excluded. Metadata labels were harmonized with the final assignments before analysis.

To place the CKC within the genus, the retained genomes were compared with a representative *Corynebacterium* genome collection using fastANI. The ten nearest non-CKC genomes were retained as phylogenomic outgroups, and the five closest representatives were displayed in the ANI heatmap. Digital DNA–DNA hybridization (dDDH) estimates were generated using the Genome-to-Genome Distance Calculator (GGDC 3.0) with Formula 2 (Auch et al., 2010). Comparisons were performed between the 56 retained genomes and the reference genomes of *C. kroppenstedtii* DSM 44385 (GCF_000023145.1), *C. parakroppenstedtii* MC-26 (GCF_019754935.1), and *C. pseudokroppenstedtii* P15_C1 (GCF_030486685.2). A dDDH value of ≥70% was used as the threshold for species-level relatedness.

### Genome annotation and pangenome construction

Genomes were annotated by using Bakta v1.12.0 with the full database (Schwengers et al., 2021). Average amino acid identity (AAI) was assessed using FastAAI with predicted protein sets generated by Bakta (Gerhardt et al., 2025). PGAP2 was adopted to construct the pan-genome for CKC with the strict mode (Bu et al., 2025). Briefly, PGAP2 used MMseqs2 v18.8cc5c for clustering and DIAMOND v2.2.4 for alignment, with orthologue, paralogue, and duplication thresholds of 0.98, 0.70, and 0.99 (Buchfink et al., 2015; Steinegger and Söding, 2017). Clusters were assigned to four frequency classes: strict core (100% of genomes), soft core (95 to <99%), shell (15 to <95%), and cloud (<15%). We defined strict and soft core together as the core genome, and shell and cloud together as the accessory genome.

PGAP2 post-processing generated accumulation curves for the pangenome and core genome. We fitted a power-law model to pangenome growth and an exponential-decay model to core-genome size. A positive power-law exponent below one was taken as evidence of an open pangenome (Kim et al., 2020). Cluster copy numbers were converted to binary presence or absence for gene-content analyses.

To evaluate the sensitivity of pangenome inference to assembly quality, we repeated the PGAP2 analysis after restricting the dataset to genomes that met all pre-specified assembly-quality criteria: genome fraction ≥95%, ≤200 contigs, ≤400 mismatches per 100 kb, and ≤5 misassemblies. The restricted analysis used the same Bakta annotations, PGAP2 version, and strict-mode settings as the primary analysis. Pangenome partitions and accumulation-curve parameters were then compared between the full dataset and the QC-restricted dataset.

### Core-genome phylogeny and recombination analysis

The selected outgroups were annotated with Bakta and included with the CKC genomes in a strict-mode PGAP2 analysis. Core genes present in at least 80% of taxa were aligned with MAFFT (Katoh and Standley, 2013). A maximum-likelihood tree was inferred with IQ-TREE v3.1.2 under GTR + F + G4 with 1,000 ultrafast bootstrap replicates (Minh et al., 2020).

Lineage concordance within the CKC was examined in a separate core-gene alignment. Regions identified as recombinant by Gubbins were removed before phylogenetic analysis (Croucher et al., 2015). The filtered polymorphic sites were analysed with IQ-TREE under TVM + F + ASC with 1,000 ultrafast bootstrap replicates. Pairwise cgSNP distances were calculated from the same filtered alignment with snp-dists.^1^ Fine-scale groups were interpreted only when recovered by both the principal and recombination-filtered trees.

### Accessory genome ordination and population structure

Accessory genome ordination used non-singleton clusters, thereby reducing the contribution of isolate-specific and assembly-specific variation. Jaccard dissimilarities from the binary matrix were summarized by PCoA based on R software v4.5.2. Species differences were tested by PERMANOVA with 999 permutations (Anderson, 2001). PERMDISP assessed differences in within-species dispersion from distances to group centroids in the positive PCoA coordinate space, using the same permutation scheme. Within Cpak, population structure was further resolved by partitioning around medoids (PAM) of the Jaccard distance matrix derived from accessory-gene presence/absence profiles.

### Identification of lineage-associated accessory clusters

For each lineage or species comparison, cluster enrichment was tested against the relevant background by one-sided Fisher exact test with R. *p* values were adjusted within each comparison by the Benjamini–Hochberg method. High-confidence candidates were shell or cloud clusters present in at least two target genomes. They also required at least 70% target prevalence, no more than 20% background prevalence, and adjusted *p* ≤ 0.05. Clusters found in every target genome and no background genome were classified as group restricted. We compared each Cpak lineage with the remaining Cpak genomes and performed reciprocal comparisons between Cpak and Cpse.

To evaluate the predictive robustness of lineage-associated accessory-cluster panels, internal repeated stratified cross-validation was performed for the three dataset-defined Cpak lineages with in-house R script. In each split, candidate accessory clusters were re-selected using the training genomes only according to the same prevalence- and enrichment-based criteria applied in the primary analysis. The resulting lineage-associated cluster panels were used to classify held-out genomes. Performance was summarized by sensitivity, specificity, precision, F1 score, balanced accuracy, and the aggregated confusion matrix.

### Functional annotation and enrichment analysis

Representative cluster proteins were annotated with eggNOG-mapper v2 (Cantalapiedra et al., 2021). We combined these assignments with Bakta product descriptions, SignalP v6.0 predictions (Teufel et al., 2022), and TMHMM topology predictions (Krogh et al., 2001). Predefined features covered COG classes, transport functions, signal peptides, lipoprotein signals, transmembrane topology, and cell-envelope or lipid-related annotations.

Each prioritized cluster set was compared with the remaining annotated pangenome by Fisher exact test. Benjamini–Hochberg correction was applied across all group–feature combinations. Biological interpretation was limited to mechanism-relevant features with global FDR < 0.05; annotation-coverage indicators were not interpreted. Separately, prevalence and neighbourhood conservation of a pre-specified lipid- and iron-related cluster set were compared across species and Cpak lineages using assembly-contiguous representatives. Neighbourhood links denote shared PGAP2 cluster identity.

### Mobile genetic element detection and MGE–pangenome overlap

geNomad v1.12.0 predicted plasmid-like and viral sequences (Camargo et al., 2023), while ISEScan v1.7.3 identified ISs (Xie and Tang, 2017). We summarized IS elements and families, plasmid-like sequences, proviruses, and total viral sequences for each genome. IS burden was the proportion of assembled sequence covered by predicted IS elements. MOB-suite v3.1.9 was run independently on each assembly to reconstruct putative plasmid contigs and report primary plasmid clusters, replicon and relaxase types, predicted mobility and host range, mating-pair formation systems, and *oriT* sites (Robertson and Nash, 2018). geNomad plasmid-like sequence counts remained the primary measure in group comparisons. MOB-suite calls were used for cross-tool comparison, plasmid cluster classification, and the plasmid-contig component of the coordinate-overlap analysis. These calls were treated as sequence-based predictions rather than evidence of autonomous plasmids or transfer.

Predicted IS, provirus, and plasmid-contig intervals were integrated for coordinate analysis. MOB-suite contigs reported as plasmid molecules were represented by their full contig spans. Raw contigs were matched to Bakta/PGAP2 identifiers by MD5 identity of upper-case nucleotide sequences. PGAP2 sample indices came from the gene-content header, and gene indices were linked to Bakta CDS features ordered by genomic start. Paralogous member genes were expanded before overlap testing. A cluster was positive when any member CDS intersected a predicted MGE by at least 1 bp. Frequency classes were compared by chi-square test, and accessory versus core clusters by Fisher’s exact test. Requiring at least 50% CDS overlap provided a sensitivity analysis. We additionally conducted a cluster-level sensitivity analysis adjusting for log(member-gene count).

### Antimicrobial-resistance genes and AC410-like elements

ARGs were identified against CARD database (Alcock et al., 2023). The main analysis retained matches with at least 80% sequence identity and 80% reference coverage, then summarized them by genome and PGAP2 cluster.

We combined MOB-suite classification and complete-genome sequence similarity as two sources of AC410-like evidence. Contigs assigned to the AC410 or related AC411 primary cluster were retained as MOB-suite evidence. Complete CKC genomes were also searched with BLASTn against the AC410 reference to identify chromosomal homologues that draft-plasmid reconstruction might miss (Boratyn et al., 2013). Association with carriage of a CARD-defined ARG was tested by two-sided Fisher exact test, and group proportions received exact binomial confidence intervals. Relaxases, mating-pair formation genes, *oriT* sites, and recognized replicons were summarized from MOB-suite output. Sequence classification or similarity was not treated as evidence of autonomous replication, excision, or transfer.

### Comparative analysis of AC410-like regions

The chromosomal AC410-like locus in Cpak genome SAMN57172177 was compared with *C. resistens* plasmid pJA144188 and homologous chromosomal regions from other *Corynebacterium* species. Pairwise BLASTn links required at least 90% nucleotide identity across 500 bp. Bakta, CARD, ISEScan, and MOB-suite annotations were integrated to distinguish ARGs, IS or transposase genes, the integron integrase, replication or mobility genes, other annotated genes, and hypothetical proteins.

### Statistical analysis

Tests were two sided unless a directional enrichment hypothesis was specified. Continuous traits across species or lineage groups were compared by Kruskal–Wallis test, and genome-level associations by Spearman rank correlation. Ordinary least-squares lines in figures served only as visual summaries. For the IS–accessory association, we calculated a partial Spearman correlation after residualizing the ranks of IS count and accessory-cluster count against assembled genome size (Mb). We additionally assessed robustness after controlling for log10(contig count + 1), after restriction to QC-keep genomes, and within Cpak after controlling for genome size and PAM lineage. Categorical data were analysed by chi-square or Fisher exact test as appropriate, with Benjamini–Hochberg correction for multiple comparisons. For the CDS-instance overlap analysis, odds ratios and 95% confidence intervals were obtained from logistic regression with genome fixed effects and PGAP2-cluster-robust standard errors. This analysis was performed separately for the ≥1-bp and ≥50%-CDS overlap definitions.

## Results

### Genome reassignment resolves three species within a well-delimited CKC

We first re-evaluated the taxonomic composition and assembly quality of public genomes assigned to the CKC. Of the 71 initial assemblies, 13 fell below 95% ANI to all three CKC references and two failed the coverage and fragmentation criteria. The final dataset (*n* = 56) comprised two Ck, 47 Cpak, and seven Cpse genomes. ANI reassignment changed the species labels of seven retained records (Supplementary Table S1). Eight genomes were complete, but contiguity varied widely across the collection. Genome sizes ranged from 2.438 to 2.963 Mb, GC content from 55.66 to 57.46%, and contig counts from 1 to 376.

**Figure 1:**
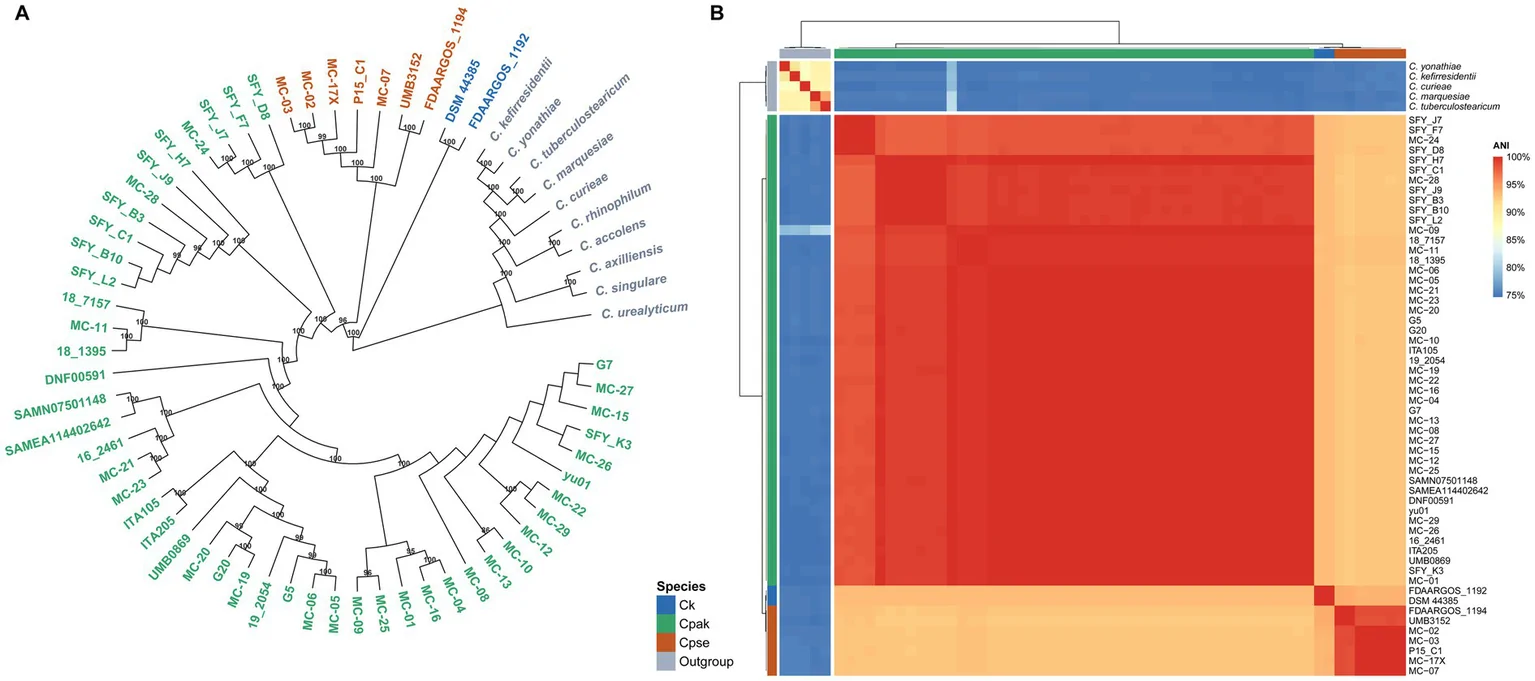
Genome-based delimitation and phylogenetic placement of the CKC **(A)** Maximum-likelihood phylogeny inferred from a 1,928,988-nt concatenated core-gene alignment of 56 CKC genomes and the ten nearest non-CKC *Corynebacterium* representatives selected by ANI. Branch lengths are suppressed in the display; bootstrap values of at least 70% are shown, and tip colours denote *Ck*, *Cpak*, and *Cpse*. **(B)** ANI heatmap for the 56 CKC genomes and five closest outgroups. Rows and columns are hierarchically clustered and annotated by final species assignment.

The CKC was clearly separated from the 183 representative *Corynebacterium* genomes. The highest ANI to a non-CKC representative was 80.87%, whereas every retained genome shared at least 98.16% ANI with its assigned CKC reference (Figure 1). Formula 2 dDDH values were ≥70% only for comparisons with the reference strain of the ANI-assigned species. The two Ck genomes each showed 100.0% dDDH relative to DSM 44385, whereas values for Cpak and Cpse ranged from 80.9 to 100.0% and from 84.9 to 100.0%, respectively. In contrast, between-species dDDH values ranged from 45.2 to 51.9%, with all 95% confidence intervals below 70% (Supplementary Table S1). AAI analysis also showed higher mean Jaccard similarity of shared single-copy proteins within species (0.9869–0.9996) than between species (0.9168–0.9246). The 66-taxon core-genome phylogeny placed all outgroups outside the CKC, while Ck, Cpak, and Cpse each formed a monophyletic group, with Cpak and Cpse recovered as sister taxa. Collectively, these analyses supported the three-species framework used in the subsequent analyses.

### The CKC has an open pangenome dominated by accessory clusters

**Figure 2:**
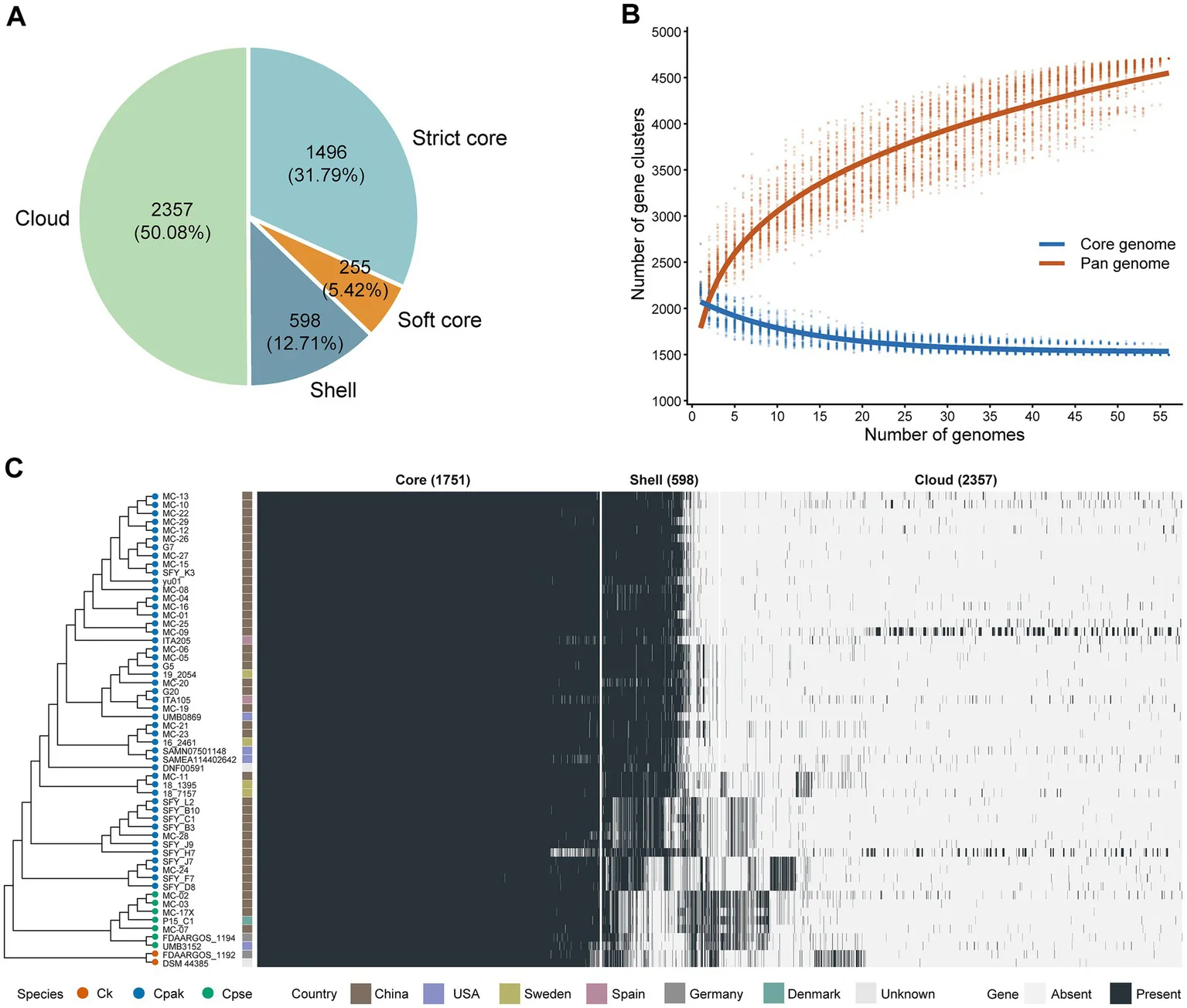
Pangenome composition and gene presence/absence structure of the CKC **(A)** Distribution of the 4,706 PGAP2 gene clusters among strict-core, soft-core, shell, and cloud partitions. **(B)** Core- and pangenome accumulation curves across genome resampling, with the fitted exponential core-genome curve and power-law pangenome curve. **(C)** Presence/absence matrix of all 4,706 clusters, with genomes ordered by the CKC core-gene phylogeny and clusters grouped into core (strict plus soft core; *n* = 1,751), shell (*n* = 598), and cloud (*n* = 2,357) partitions. The annotation strip shows country metadata; these annotations are descriptive because geographical sampling was unbalanced.

With the species assignments established, we examined how much gene content was shared across the complex and whether the pangenome approached saturation. PGAP2 identified 4,706 gene clusters, of which 1,496 were strict core, 255 soft core, 598 shell, and 2,357 cloud (Figure 2A). The accessory genome contained 2,955 clusters, representing 62.79% of the pangenome. Half of all clusters occurred in fewer than 15% of genomes.

The pangenome growth exhibited a typical open architecture, following the power-law function *y* = 1788.064 × *x*^0.232^ (Figure 2B). This trajectory suggests that sequencing additional genomes will continue to reveal novel gene families. When genomes were ordered by the core-gene phylogeny, shell and cloud presence–absence profiles changed broadly across the Ck, Cpak, and Cpse branches (Figure 2C). This correspondence showed that species-level accessory genome composition was consistent with the classification recovered from the core genome. Moreover, geographic interpretation of the Cpak-Cpse species split was limited by the predominance of Chinese genomes.

To assess the robustness of pangenome inference to assembly quality, we repeated the analysis using a QC-restricted set of 38 genomes that satisfied all pre-specified assembly-quality thresholds: genome fraction ≥95%, ≤200 contigs, ≤400 mismatches per 100 kb, and ≤5 misassemblies. This subset comprised two Ck, 31 Cpak, and five Cpse genomes. This analysis identified 3,533 gene clusters, comprising 1,739 strict-core, 78 soft-core, 510 shell, and 1,206 cloud clusters. Compared with the full dataset, the proportion of cloud clusters was lower in the QC-restricted analysis (34.14% versus 50.08%). Nevertheless, the pangenome accumulation curve remained consistent with an open pangenome, with a positive fitted exponent (*y* = 2013.472 × *x*^0.149^; Supplementary Figure S2). These results indicate that the overall open-pangenome pattern was retained after restriction to higher-quality assemblies, whereas the estimated abundance of cloud clusters was sensitive to assembly quality and dataset composition.

### Accessory gene content separates species and resolves three Cpak lineages

**Figure 3:**
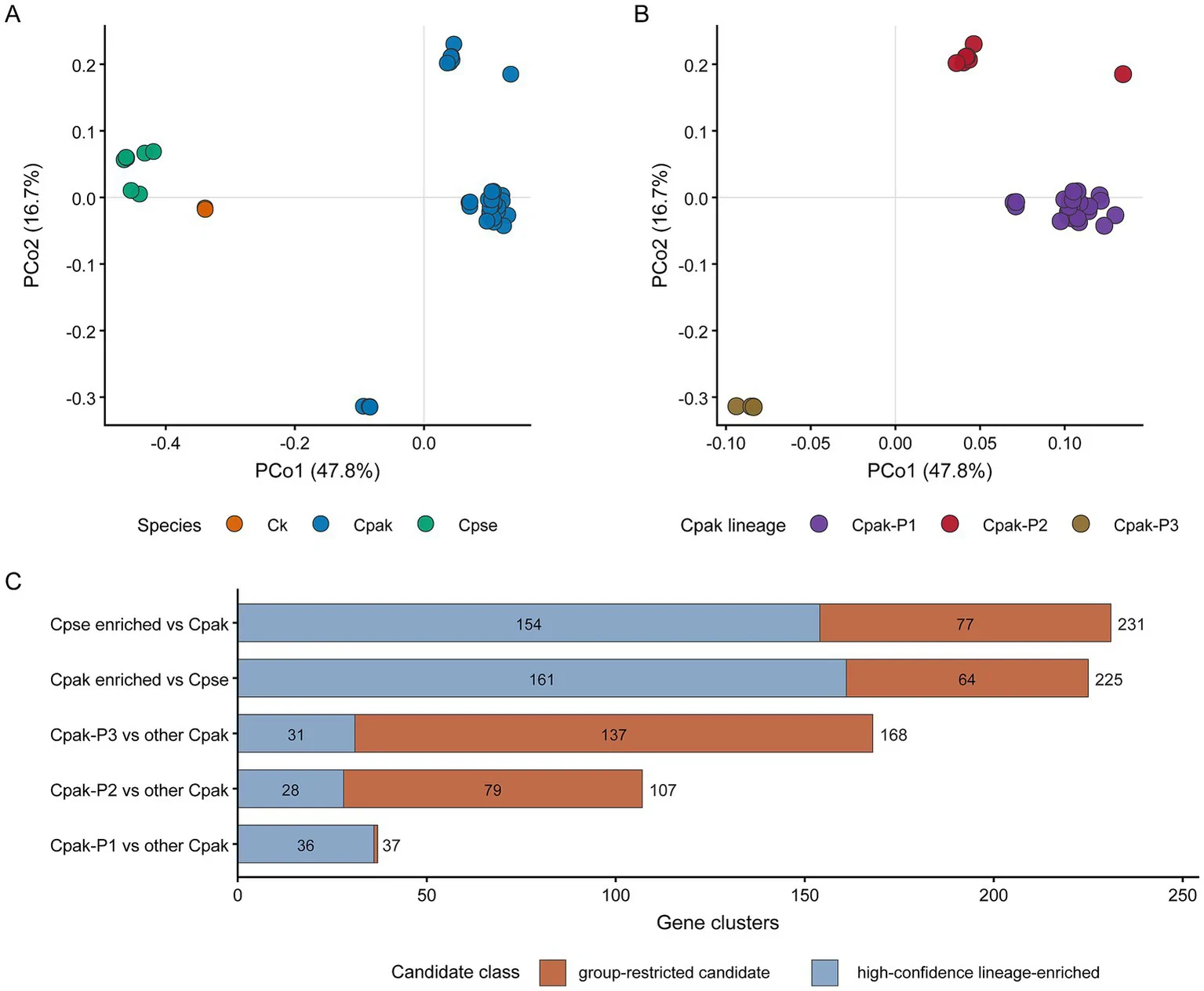
Accessory genome population structure and lineage-associated gene clusters. **(A)** Principal coordinates analysis of binary Jaccard dissimilarities among 56 genomes using 1,596 clusters present in at least two genomes. Points are coloured by species. Species identity explained 54.2% of the variation by PERMANOVA (*p* = 0.001), while PERMDISP did not detect a significant difference in within-species dispersion (*p* = 0.397). **(B)** The Cpak subset coloured by the three-group PAM solution (Cpak-P1 to Cpak-P3). **(C)** Numbers of prioritized lineage-associated accessory clusters for Cpak-P1, Cpak-P2, and Cpak-P3 and for reciprocal Cpak–Cpse comparisons. Bars distinguish group-restricted candidates from high-confidence enriched candidates.

The non-singleton accessory genome was analysed to assess whether gene content variation reflected the established species boundaries and captured additional structure within Cpak. PCoA of 1,596 clusters separated the three species along axes explaining 47.79 and 16.66% of the variation (Figure 3A). Species identity accounted for 54.2% of Jaccard-distance variation (PERMANOVA *R*^2^ = 0.542, *F* = 31.366, *p* = 0.001). PERMDISP was not significant (*F* = 2.074, *p* = 0.397), providing no evidence that unequal dispersion accounted for the separation. This interpretation remains limited for Ck because only two genomes were available. Within Cpak, the best-supported PAM solution contained three groups (silhouette width, 0.671): Cpak-P1 (*n* = 36), Cpak-P2 (*n* = 7), and Cpak-P3 (*n* = 4) (Figure 3B).

The same groups were recovered in the core phylogeny after recombination filtering (Supplementary Figure S3). Gubbins predicted 517 recombination blocks, with 24,050 SNPs assigned to these blocks. Median within-lineage cgSNP distances were 148, 36, and 338 for Cpak-P1, Cpak-P2, and Cpak-P3, respectively. The median distance between Cpak-P1 and Cpak-P2 was 579 cgSNPs, whereas Cpak-P3 was separated from Cpak-P1 and Cpak-P2 by 8,297 and 8,419 cgSNPs, respectively. This concordance supported the interpretation that the accessory genome groups represented broader genomic lineages rather than patterns restricted to a single data type.

Following delineation of Cpak-P1, Cpak-P2, and Cpak-P3, lineage-associated accessory clusters were identified (Supplementary Table S2). We identified 37, 107, and 168 clusters associated with Cpak-P1, Cpak-P2, and Cpak-P3, respectively (Figure 3C). Among these, 1, 79, and 137 were restricted to the corresponding lineage. Reciprocal species comparisons found 225 clusters enriched in Cpak and 231 enriched in Cpse, including 64 and 77 group-restricted clusters. The large restricted sets in Cpak-P2 and Cpak-P3 indicated marked gene-content differentiation within Cpak.

Internal repeated stratified cross-validation further supported the reproducibility of these lineage-associated accessory-cluster panels. Across 100 repeated four-fold runs, with candidate clusters re-selected in each training fold, all held-out Cpak genomes were assigned to their original Cpak-P1, Cpak-P2, or Cpak-P3 working lineage; mean overall accuracy, macro-F1 score, and macro balanced accuracy were all 1.000 (Supplementary Table S2). These results support the internal consistency of the candidate panels, although estimates for Cpak-P3 should be interpreted cautiously because of its limited sample size.

### Mobile genetic elements are lineage structured and associated with accessory genome variation

**Figure 4:**
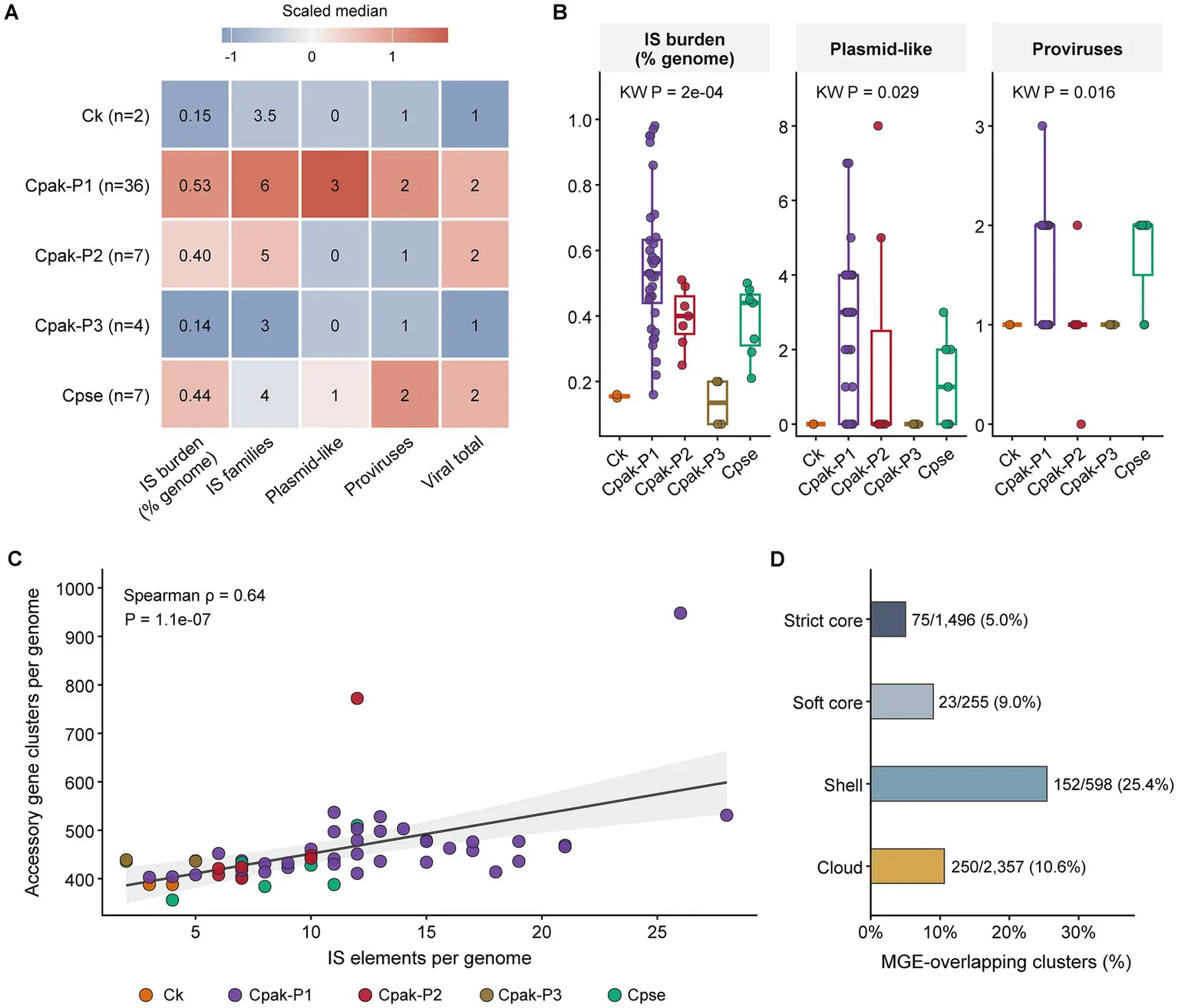
Mobile genetic element profiles and accessory genome divergence in the *Corynebacterium kroppenstedtii* complex. **(A)** Species- and lineage-level MGE profiles. Tile colours show column-standardized group medians, and values show unscaled medians; *n* denotes genomes. Plasmid-like sequences, proviruses, and total viral sequences were predicted with geNomad, whereas insertion sequences (ISs) were identified with ISEScan. **(B)** Distributions of IS burden, plasmid-like sequences, and proviruses across Ck, Cpak-P1, Cpak-P2, Cpak-P3, and Cpse. Points represent genomes and boxes show the median and interquartile range; *P* values are from two-sided Kruskal–Wallis tests. **(C)** Association between IS count and accessory gene cluster count. The line and shading show an ordinary least-squares visual trend and 95% confidence band; inference is based on a two-sided Spearman correlation across all 56 genomes. **(D)** Proportions of gene clusters with at least one member CDS overlapping a predicted MGE by ≥1 bp. Labels show MGE-overlapping clusters divided by all clusters in each partition.

To examine whether MGEs accompanied this accessory genome structure, we compared element burdens across groups and tested their relationship with gene-content variation. MGE profiles varied across species and Cpak lineages (Figures 4A,B). Cpak-P1 had the highest median IS burden at 0.53% of assembled sequence. Group differences were detected for IS burden (Kruskal–Wallis *p* = 1.99 × 10^−4^), plasmid-like sequences (*p* = 0.0291), and proviruses (*p* = 0.0157).

Across all 56 genomes, IS count correlated with accessory gene cluster count (Spearman’s *ρ* = 0.640, *p* = 1.12 × 10^−7^; Figure 4C). After adjustment for assembled genome size, the association remained positive (partial Spearman *ρ* = 0.397, *p* = 0.0027; 10,000-permutation *p* = 0.0021; Supplementary Figure S4). The result was similar after additional adjustment for contig count ( ρ = 0.407, *p* = 0.0022), in the QC-keep subset (38 genomes; *ρ* = 0.448, *p* = 0.0054), and within Cpak after adjustment for genome size and PAM lineage (47 genomes; *ρ* = 0.449, *p* = 0.0023).

Of 811 predicted MGE intervals, 747 overlapped at least one CDS. MGE overlap was detected in 75/1,496 strict-core, 23/255 soft-core, 152/598 shell, and 250/2,357 cloud clusters (Figure 4D). Overall differences among the four classes were significant (Chi-square = 188.11, df = 3, *p* = 1.56 × 10^−40^). Accessory clusters were more likely to overlap an MGE than core clusters (402/2,955 versus 98/1,751; odds ratio = 2.66, *p* = 3.31 × 10^−19^). At the CDS-instance level, MGE overlap was more frequent among accessory than core CDSs (2,143/25,778 [8.31%] versus 535/97,770 [0.55%]). After adjusting for CDS length and genome fixed effects, accessory CDSs remained more likely to overlap an MGE (OR = 15.88, 95% CI 10.21–24.71; *p* = 1.41 × 10^−34^). This result was retained using the stricter ≥50%-CDS overlap definition (OR = 15.32, 95% CI 9.78–23.99; *p* = 8.88 × 10^−33^; Supplementary Table S3). Collectively, these analyses support an association between MGE-related measures and accessory genome variation, but do not distinguish MGE effects from other processes underlying pangenome openness.

### AC410-like regions are associated with ARG carriage

**Figure 5:**
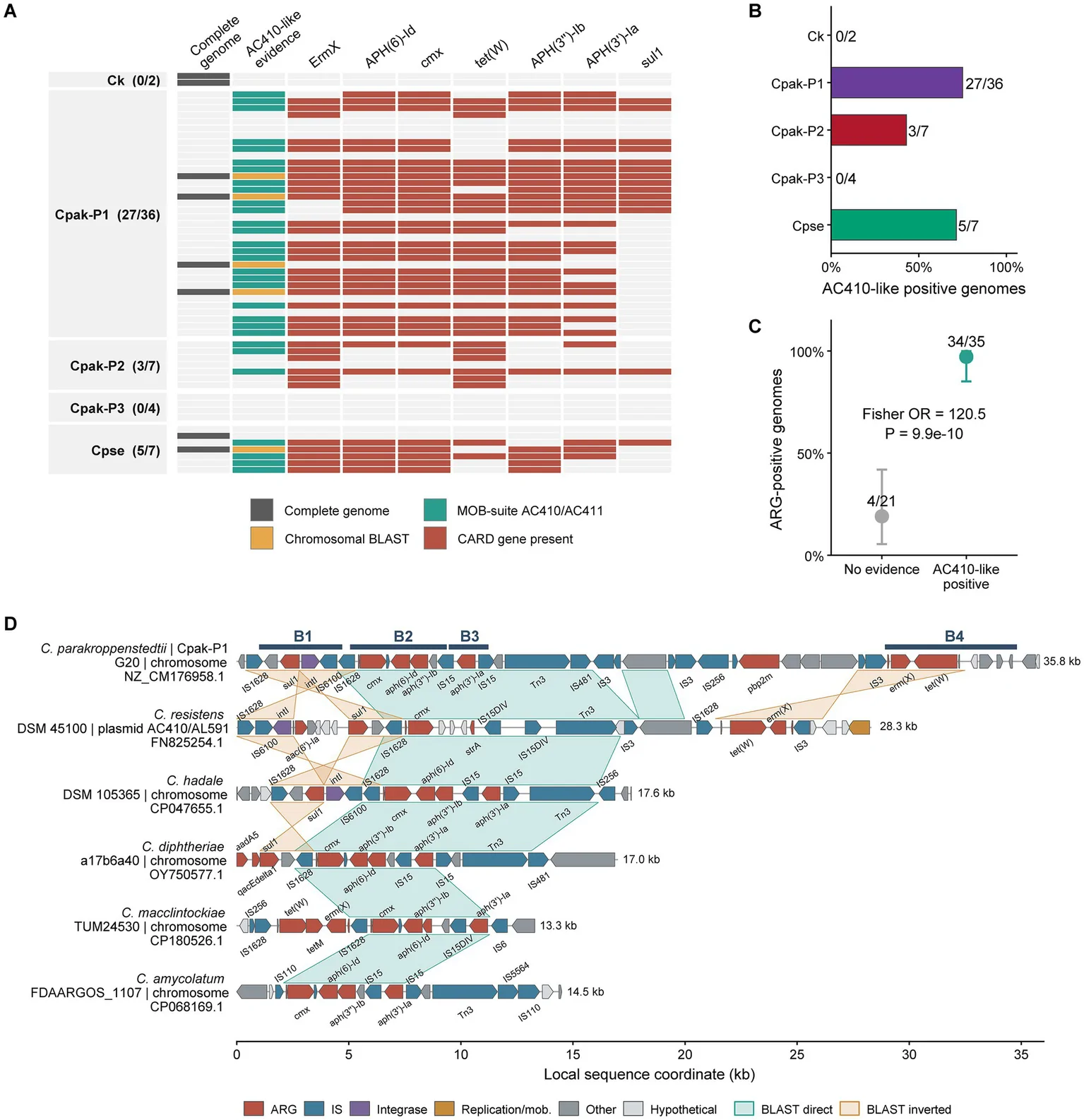
Distribution, antimicrobial-resistance association, and comparative context of AC410-like elements. **(A)** Sample-level distribution of AC410-like evidence and seven CARD 80/80 ARGs across 56 CKC genomes. Genomes are grouped by species or Cpak lineage and ordered within groups according to the core-gene phylogeny. AC410-like evidence comprises MOB-suite AC410/AC411 assignments in 30 draft genomes and chromosomal BLAST evidence in five additional complete genomes; parentheses denote positive genomes divided by all genomes in the group. **(B)** Lineage prevalence of combined AC410-like evidence. **(C)** Association between AC410-like evidence and ARG carriage. Points show ARG-positive proportions with exact binomial 95% confidence intervals. The odds ratio and *P* value are from a two-sided Fisher exact test; 34/35 AC410-like-positive genomes and 4/21 genomes without AC410-like evidence carried at least one CARD gene. **(D)** Comparative organization and pairwise synteny of the chromosomal AC410-like locus and homologous regions in five complete *Corynebacterium* sequences. The 35.8-kb Cpak SAMN57172177 locus is compared with the 28.3-kb *C. resistens* plasmid pJA144188 (MOB-suite AC410/AL591) and local chromosomal neighbourhoods from *C. hadale*, *C. diphtheriae*, *C. macclintockiae*, and *C. amycolatum*. Arrows indicate coding sequences and orientation; colours distinguish ARGs, IS or transposase genes, the integron integrase, replication or mobility genes, other annotated genes, and hypothetical proteins. Ribbons connect nonredundant pairwise BLAST matches between adjacent sequences (identity ≥90%; alignment length ≥500 bp). B1–B4 mark four AC410-reference BLAST blocks.

The potential link between plasmid-related regions and antimicrobial resistance was examined by testing whether AC410-like evidence was associated with ARG carriage. Combined MOB-suite and complete-genome BLAST analyses detected AC410-like sequences in 35 of 56 genomes (Figure 5A). AC410-like evidence was frequent in Cpak-P1 (27/36) and Cpse (5/7), less common in Cpak-P2 (3/7), and absent from the sampled Cpak-P3 and Ck genomes (Figure 5B). Among the 30 MOB-suite assignments, 29 agreed with geNomad plasmid-like predictions. Twenty-seven assignments, comprising 26 AC410 and one AC411 assignment, carried a MOBP-family relaxase and were classified as mobilizable, whereas three AC410 assignments were classified as non-mobilizable; none contained a detected mating-pair formation system, *oriT*, or recognized replicon. All 29 AC410 assignments had a genus-level predicted host range within the genus *Corynebacterium*, whereas the AC411 assignment had a broader predicted host range.

Across the 35 genomes carrying AC410-like sequences, seven distinct ARGs were detected. *ErmX* was present in 35 genomes, *cmx* and *APH(6)-Id* in 33 each, and *tet(W)* in 31. *APH(3″)-Ib*, *APH(3′)-Ia*, and *sul1* occurred in 30, 27, and 14 genomes, respectively. At least one ARG occurred in 34/35 AC410-like-positive genomes, compared with 4/21 negative genomes (Figure 5C). This corresponded to an odds ratio of 120.515 (95% confidence interval, 13.511–5905.055; *p* = 9.93 × 10^−10^). In the complete chromosome of *C. parakroppenstedtii* SAMN57172177, five ARG annotations, *sul1*, *cmx*, *aph(6)-Id*, *aph(3″)-Ib* and *aph(3′)-Ia*, overlapped at least one AC410-reference BLAST block within the 35.8-kb comparative locus (Figure 5D). The pbp2m annotation lay within the same comparative locus but outside the four AC410-reference blocks.

Among the eight complete genomes, five carried AC410-like sequences on their sole chromosome rather than on an independent plasmid contig (Supplementary Figure S5). Comparative synteny showed that the 35.8-kb locus in SAMN57172177 shared conserved blocks with *C. resistens* plasmid pJA144188 and chromosomal regions in four other *Corynebacterium* species (Figure 5D) (Schröder et al., 2012). These observations provide direct evidence of physical colocalization for specific ARGs in a representative complete CKC genome. However, they do not imply that every ARG in all AC410-like-positive genomes is physically linked to the region, or establish autonomous replication, excision, transfer, or evolutionary origin.

### Functional divergence identifies a candidate lipid–iron locus

**Figure 6:**
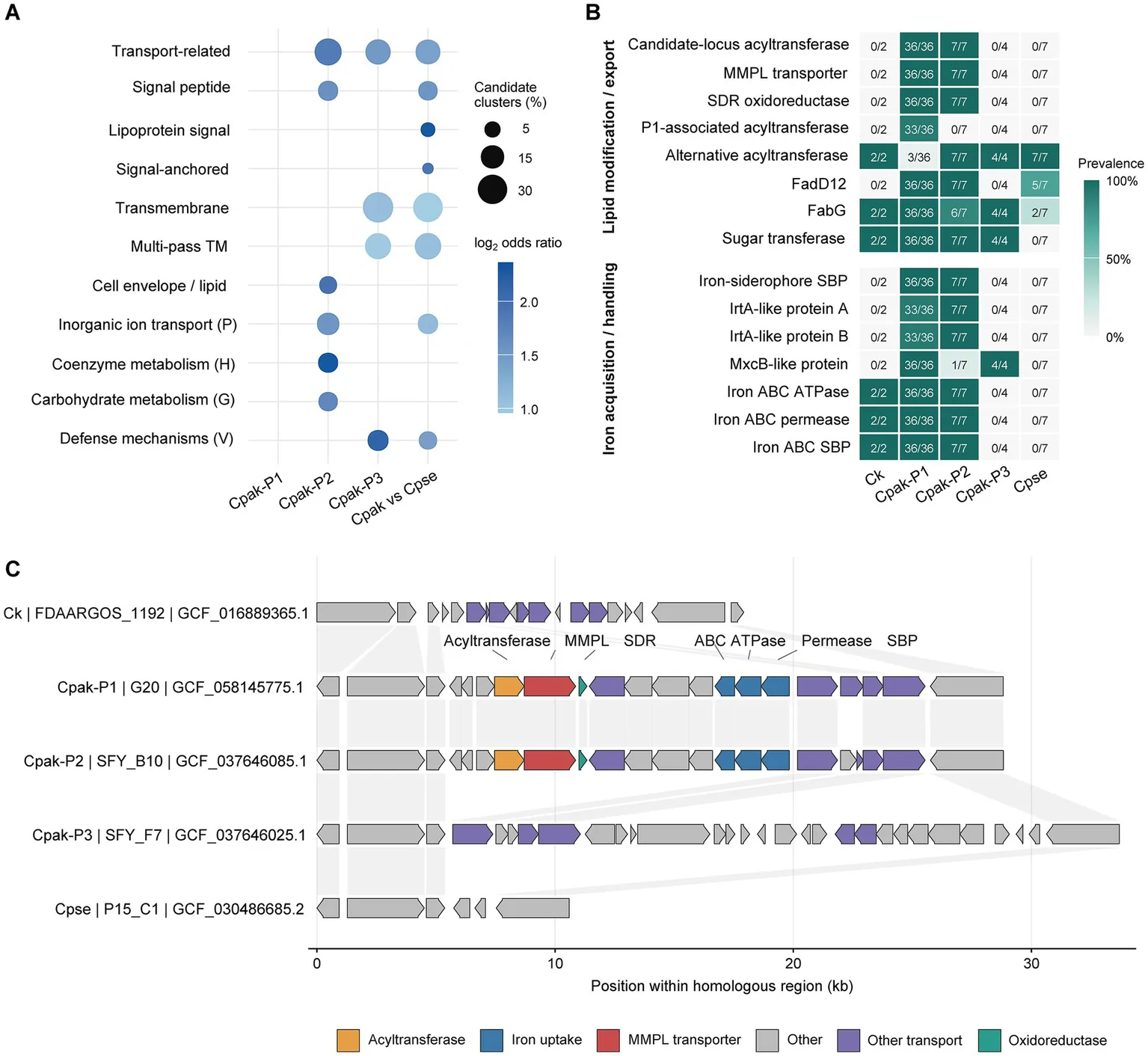
Functional divergence and a candidate lipid–iron locus across the CKC. **(A)** Functional features enriched among prioritized lineage-associated accessory clusters. Only mechanism-relevant features with global Benjamini–Hochberg FDR < 0.05 are shown. Point size denotes the proportion of candidate clusters carrying the feature and colour denotes the log2 odds ratio. The absence of points for Cpak-P1 indicates that no selected feature passed the global FDR threshold. **(B)** Prevalence of 15 pre-specified clusters related to lipid modification or export, sugar transfer, and iron acquisition or handling across Ck, Cpak-P1, Cpak-P2, Cpak-P3, and Cpse. Values denote positive genomes divided by all genomes in each group. **(C)** Homologous gene neighbourhoods bounded by two strict-core anchor clusters in representative genomes. Genes are coloured by predicted function; grey ribbons connect shared PGAP2 orthologous clusters. The acyltransferase–MMPL–oxidoreductase segment and adjacent iron-uptake genes form a candidate lipid–iron locus conserved in Cpak-P1 and Cpak-P2. Comparative annotation does not establish corynekropbactin biosynthesis.

Functional enrichment was most pronounced in Cpak-P2 and Cpak-P3 (Figure 6A). Cpak-P2-associated clusters were enriched for transport (25/107; odds ratio = 3.77, FDR = 2.52 × 10^−5^). Coenzyme transport and metabolism was also enriched (COG H; 10/107; odds ratio = 5.13, FDR = 0.00171). Inorganic-ion transport and metabolism showed a similar pattern (COG P; 14/107; odds ratio = 3.05, FDR = 0.00781). Cpak-P3 showed enrichment for transport, defense, and membrane-related functions, including 55/168 transmembrane-protein clusters (odds ratio = 2.17, FDR = 2.65 × 10^−4^).

Family-level classification showed that ABC-system components predominated among transport-related candidates in both Cpak-P2 (17/25) and Cpak-P3 (21/32) (Supplementary Table S4). The remaining Cpak-P2 candidates included MFS transporters (3/25), PTS components (2/25), one energy-coupling factor component, and one cation diffusion facilitator; one multi-pass membrane protein could not be resolved beyond a transport-related annotation. Cpak-P3 additionally contained ABC-associated FtsX/MacB components (3/32), MFS transporters (3/32), and single PTS, energy-coupling factor, DMT/EamA, sodium solute symporter, and TauE/SafE-family candidates. Thus, the enrichment signal reflected diverse predicted transport functions rather than expansion of a single transporter family. No selected Cpak-P1 feature passed the global FDR threshold.

In a separate pre-specified analysis, we examined 15 clusters related to lipid handling and iron acquisition or transport. An acyltransferase–MMPL transporter–short-chain dehydrogenase/reductase segment occurred in all Cpak-P1 (36/36) and Cpak-P2 (7/7) genomes but was absent from Cpak-P3, Ck, and Cpse (Figure 6B). Related siderophore-, iron-, and ABC-transport-associated modules showed similar lineage structure. The interval retained the same strict-core anchors in assembly-contiguous representatives, with broader neighbourhood conservation in Cpak-P1 and Cpak-P2 (Figure 6C). Its conserved distribution and gene order supported its designation as a candidate lipid–iron locus, although its biochemical product and pathogenic relevance remain unknown.

## Discussion

Whether the CKC can be treated as a single genomic and clinical entity has remained uncertain because many isolates were named before Cpak and Cpse were recognized. Our analysis shows that this simplification obscures substantial structure. ANI and core-gene phylogeny separated Ck, Cpak, and Cpse, while core and accessory variation consistently resolved three working lineages within Cpak. Cpak-P3 formed a relatively deep group, whereas Cpak-P2 remained close to Cpak-P1. The uneven distribution of ARG-associated regions and candidate metabolic loci suggests that historical series based on legacy Ck labels may combine biologically distinct organisms (Luo et al., 2022; Huang et al., 2024). Genome-resolved identification should therefore precede attempts to associate CKC isolates with disease phenotype or treatment response. The Cpak lineages remain dataset-defined genomic groups rather than formal taxa or epidemiological clones, particularly given the small Cpak-P3 sample. Although internal cross-validation supports the reproducibility of the lineage-associated accessory-cluster panels, validation in an independent CKC collection will be required before their broader diagnostic or epidemiological utility can be established.

A related evolutionary question is why gene content varies so widely across the complex. The open pangenome implies continued gene-family discovery, but its fitted exponent does not identify the underlying mechanism. Concordance between core and accessory structure indicates that population history contributes substantially to this variation (Kim et al., 2020). Similar combinations of inherited structure and gene acquisition have been described elsewhere in the genus. The distributed genome of *C. diphtheriae* includes horizontally acquired pathogenicity islands, whereas *C. pseudotuberculosis* biovars differ in genome plasticity (Trost et al., 2012; Soares et al., 2013). Studies of the open pangenomes of *C. striatum* and *C. amycolatum* described source-related diversity in the former and resistance-associated genomic islands in the latter (Jesus et al., 2022; Qiu et al., 2023). Extending this comparative perspective, a recent genome-scale analysis of β-lactamase-producing *Klebsiella pneumoniae* integrated pangenome heterogeneity with resistome, virulome, and mobilome profiles (Biswas and Anbarasu, 2026). This approach provides a useful framework for considering how genomic plasticity, MGEs, and ARG carriage may co-occur in the CKC.

In the CKC, the open-pangenome pattern persisted in the QC-restricted PGAP2 analysis, although the cloud fraction declined from 50.08% in the full dataset to 34.14% in the 38-genome subset. This shift indicates that cloud gene cluster abundance should be interpreted as a descriptive measure that remains sensitive to assembly quality and sampling. Together, the association between IS abundance and accessory gene cluster count and the excess overlap of accessory CDSs with predicted MGEs support an MGE-related component of accessory genome diversification. The CDS-level association remained after conditioning on genome-specific coding density and overlap opportunity and after accounting for CDS length. However, these adjusted statistical associations do not establish MGE activity, transfer direction, or a causal role for MGEs in pangenome openness.

Within the accessory genome, AC410-like sequences formed the most prominent resistance-associated pattern. They were concentrated in Cpak-P1 and Cpse, and genomes carrying them frequently contained determinants similar to those described in a multidrug-resistant Ck isolate and on *C. resistens* plasmid pJA144188 (Schröder et al., 2012; Fernández-Natal et al., 2016). Association at genome level, however, does not establish that every detected ARG lies within the same region. In complete CKC genomes, AC410-like sequences occurred on the chromosome. Among the MOB-suite assignments, 26/29 AC410 clusters were classified as mobilizable, and all 29 had a genus-level predicted host range within the genus *Corynebacterium*. However, no AC410/AC411 assignment contained a recognized replicon, *oriT*, or mating-pair formation system. Thus, the available data do not demonstrate autonomous replication, excision, or transfer of any AC410-like region. They instead support recurrent resistance-associated genomic backgrounds containing plasmid-related sequences, rather than a confirmed transferable plasmid or a single defined resistance island (Juhas et al., 2009; Smillie et al., 2010). Complete structural resolution and transfer experiments will be required to determine the physical linkage of individual ARGs and the mobility of these regions (Weisberg and Chang, 2023).

ARG carriage should be interpreted cautiously because antimicrobial susceptibility data for the CKC remain limited and sometimes inconsistent. *ErmX*, *tet(W)*, and *sul1* correspond to erythromycin, clindamycin, tetracycline, and trimethoprim–sulfamethoxazole resistance reported in phenotypic studies (Zhang et al., 2023). In one 90-isolate series reported as Ck, erythromycin and clindamycin resistance each reached 88.9%, while trimethoprim–sulfamethoxazole resistance reached 46.6% (Zhang et al., 2023). Those isolates were not resolved into current CKC species, so these rates cannot be assigned directly to Ck, Cpak, or Cpse. An accessory PBP2m can increase beta-lactam resistance in *C. diphtheriae* (Hennart et al., 2020), but an analogous mechanism was not tested here. The present evidence therefore does not support universal intrinsic resistance to penicillins or cephalosporins across the CKC. Notably, ARG screening cannot resolve conserved mechanisms involving penicillin-binding proteins, permeability, or cell-envelope physiology (Wang et al., 2022; Dingle et al., 2023). Species identification, ARG detection, and standardized susceptibility testing provide complementary rather than interchangeable clinical information.

Beyond antimicrobial resistance, CKC pathobiology raises the question of whether differences in metabolic capacity influence adaptation to lipid-rich, inflamed breast tissue. Cpak-P2 and Cpak-P3 differed in transport, membrane, defence, and ion-handling functions, while Cpak-P1 and Cpak-P2 shared a candidate lipid–iron locus. Its acyltransferase, MMPL-family transporter, oxidoreductase, and nearby iron-related modules generate a testable hypothesis linking lipid handling with adaptation to an inflamed, nutrient-limited environment. This possibility is relevant to the Cpak glycolipid that promotes granulomatous mastitis in experimental systems (Liu et al., 2024), but the candidate locus has not been linked to that molecule or to a disease phenotype. Our analysis likewise did not identify a dominant tox-like determinant comparable to that of toxigenic *C. diphtheriae* (Trost et al., 2012). *Cutibacterium acnes* provides a broader conceptual comparison in which lineage background and local lipid availability influence inflammatory behaviour (Fitz-Gibbon et al., 2013; Mayslich et al., 2021; Yu et al., 2024). These organisms occupy different ecological settings, and the comparison does not imply a shared mechanism. Rather, it highlights the need to examine CKC pathogenic potential through the interaction of genomic background, local metabolism, and host context.

The clinical relevance of these findings lies less in immediate treatment prediction than in defining how future CKC studies should be designed. Genomic ARG detection alone does not predict treatment response, and taxonomy alone cannot justify a beta-lactam choice or a declaration of intrinsic resistance. Expression, breakpoint selection, drug penetration, lesion physiology, drainage, and host inflammation remain important. Prospective studies should therefore pair genome-resolved identification with standardized MIC testing and clinical outcomes. Functional work could then test the structure and mobility of AC410-like regions and the biochemical role of the candidate lipid–iron locus.

Several limitations arise from the composition and quality of the public dataset. Sampling was uneven across species and regions, with Chinese genomes predominating and other locations sparsely represented; only four Cpak-P3 and two Ck genomes were available, while near-duplicate genomes limited inference about prevalence and transmission. Accordingly, the present dataset cannot support robust inference regarding geographic population structure or the geographic distribution of CKC lineages. Variation in assembly contiguity may also have affected cloud-gene counts, MGE prediction, and local gene context. Although PERMDISP found no evidence of unequal dispersion in the species comparison, the small Ck sample limits confidence. Moreover, coordinate overlap does not demonstrate mobility, and cross-sectional genomic data cannot resolve acquisition history. The absence of matched susceptibility, expression, and experimental data further restricts clinical and mechanistic interpretation. The genomic groups and candidate loci defined here therefore provide a framework for prospective validation rather than direct evidence of resistance transfer or pathogenic mechanism.

## Conclusion

The analysed CKC genomes formed three species-level groups, while Cpak contained three working lineages with distinct accessory repertoires. The pangenome was open, and MGE-related measures were associated with accessory genome variation, but these associations did not establish MGEs as a primary driver of pangenome openness. AC410-like evidence was lineage biased and strongly associated with ARG carriage, while a candidate lipid–iron locus distinguished Cpak-P1/P2 from Cpak-P3, Ck, and Cpse. These patterns provide a framework for future species-aware surveillance and mechanistic studies, but clinical, mobility, and pathogenicity claims require matched phenotypes and experimental validation.

## Data Availability

The original contributions presented in the study are included in the article/Supplementary material, further inquiries can be directed to the corresponding author.

## Footnotes

1
https://github.com/tseemann/snp-dists
